# Correlated metabolomic, genomic, and histologic phenotypes in histologically normal breast tissue

**DOI:** 10.1371/journal.pone.0193792

**Published:** 2018-04-18

**Authors:** Xuezheng Sun, Delisha A. Stewart, Rupninder Sandhu, Erin L. Kirk, Wimal W. Pathmasiri, Susan L. McRitchie, Robert F. Clark, Melissa A. Troester, Susan J. Sumner

**Affiliations:** 1 Department of Epidemiology, University of North Carolina at Chapel Hill, Chapel Hill, North Carolina, United States of America; 2 National Institutes of Health Eastern Regional Comprehensive Metabolomics Resource Core, Department of Nutrition, University of North Carolina at Chapel Hill, Nutrition Research Institute, Kannapolis, North Carolina, United States of America; 3 Lineberger Comprehensive Cancer Center, University of North Carolina at Chapel Hill, Chapel Hill, North Carolina, United States of America; 4 Analytical Chemistry and Pharmaceutics, RTI International, Durham, North Carolina, United States of America; 5 Department of Pathology and Laboratory Medicine, University of North Carolina at Chapel Hill, Chapel Hill, North Carolina, United States of America; University of South Alabama Mitchell Cancer Institute, UNITED STATES

## Abstract

Breast carcinogenesis is a multistep process accompanied by widespread molecular and genomic alterations, both in tumor and in surrounding microenvironment. It is known that tumors have altered metabolism, but the metabolic changes in normal or cancer-adjacent, nonmalignant normal tissues and how these changes relate to alterations in gene expression and histological composition are not well understood. Normal or cancer-adjacent normal breast tissues from 99 women of the Normal Breast Study (NBS) were evaluated. Data of metabolomics, gene expression and histological composition was collected by mass spectrometry, whole genome microarray, and digital image, respectively. Unsupervised clustering analysis determined metabolomics-derived subtypes. Their association with genomic and histological features, as well as other breast cancer risk factors, genomic and histological features were evaluated using logistic regression. Unsupervised clustering of metabolites resulted in two main clusters. The metabolite differences between the two clusters suggested enrichment of pathways involved in lipid metabolism, cell growth and proliferation, and migration. Compared with Cluster 1, subjects in Cluster 2 were more likely to be obese (body mass index ≥30 kg/m^2^, p<0.05), have increased adipose proportion (p<0.01) and associated with a previously defined Active genomic subtype (p<0.01). By the integrated analyses of histological, metabolomics and transcriptional data, we characterized two distinct subtypes of non-malignant breast tissue. Further research is needed to validate our findings, and understand the potential role of these alternations in breast cancer initiation, progression and recurrence.

## Introduction

Breast carcinogenesis is associated with extensive changes in the surrounding microenvironment at molecular, genomic and metabolic levels [1–4]. Our previous analysis using gene expression profiling of cancer-adjacent, nonmalignant normal tissue has defined two subtypes, termed Active and Inactive, and showed significant differences in fibrosis, wound response, and cellular adhesion [3]. Subsequent analyses further linked the Active genomic subtype to high adipose content and adverse prognosis [4, 5]. These findings underscore that considering multiple layers of data is an approach for validation, but provides novel insights into the mechanistic networks perturbed during tumorigenesis.

Although extensively studied in breast tumors, metabolic changes in surrounding microenvironment (primarily composed by histologically, nonmalignant normal breast tissue) have been less described, and little is known about how these changes relate to other level changes (e.g. gene expression) and the characteristics of tumor or patient. In this study, we hypothesized that histology, molecular pathways, and metabolic profiles vary, creating distinct niches for the development and progression of breast cancer. To test this hypothesis, we analyzed gene expression, metabolic profiles, and histological composition of normal or cancer-adjacent normal breast tissues from 99 women who participated in the Normal Breast Study (NBS) [6], particularly aimed to characterize metabolic microenvironments and correlates them to other level features.

## Materials and methods

### Study population

The Normal Breast Study (NBS) was a hospital-based study of breast cancer microenvironment and normal breast tissue, including women undergoing breast surgeries (mastectomy, lumpectomy, excisional biopsy, reduction mammoplasty, or other cosmetic breast surgery) at UNC Hospitals in Chapel Hill, North Carolina from 2009 to 2013 [6]. Fresh non-neoplastic normal breast tissues confirmed by pathology assistants at UNC Hospitals were collected at the time of breast surgery and snap-frozen in liquid nitrogen. Information on clinicopathologic, demographic, and anthropometric factors were collected from medical records and telephone interview. The present analysis included 99 women (16 with reduction mammoplasty, 83 with breast cancer). Written informed consent was obtained from all participants. Study protocols were approved by the School of Medicine Institutional Review Board at the University of North Carolina at Chapel Hill.

### Breast tissue processing

Frozen breast specimens of approximately 100 mg were prepared on dry ice and 20 μm sections were collected at both ends of the specimen and mounted on glass slides. The two sections on the ends were used for histological composition annotation, after hematoxylin and eosin (H&E) staining, and the central tissue portion was used for RNA extraction and whole genome microarray analysis. Additionally, tissues (50–120 mg) were prepared for metabolomics by gas chromatography-time of flight-mass spectrometry (GC-TOF-MS).

### Tissue composition measurement

The details regarding histological composition annotation by high-resolution digital images using the Aperio Scan-Scope XT Slide Scanner were published previously [5, 6]. Duplicate slides of same tissue specimen (one section from each end of the sample) were averaged to estimate area percentage of stroma, adipose and epithelium, given high intra-class correlation coefficients (ICCs) between replicate sections (ICC = ranged from 0.92 = 0.99).

### RNA isolation, microarrays, and Active/Inactive subtyping

The central section of fresh-frozen tissues was homogenized using a MagNA Lyser homogenizer (Roche), and RNA was isolated by QIAzol extraction followed by purification on an RNeasy column as described previously [5]. RNA quality and quantity were assessed on an Agilent 2100 Bioanalyzer and a ND-1000 NanoDrop spectrophotometer, respectively. Two-color 4 × 44 K Agilent whole-genome arrays were run on the RNA samples. Data were preprocessed before analytic statistical analyses: lowess-normalization, setting value of the probes that had a signal less than 10 dpi in either channel as missing, excluding probes that had more than 20% missing data across all samples, imputing the missing values using *k*-nearest neighbors' imputation (with k = 10), and collapsing the duplicate probes by averaging. Microarray data used in these analyses will be publicly available through the Gene Expression Omnibus (accession number GSE111601) www.ncbi.nlm.nih.gov/geo/.

Samples were classified as Active or Inactive subtype using the Creighton correlation method [5]. Briefly, a standard vector corresponding to all genes (n = 3,518) in the Active/Inactive signature was constructed, with “1” assigned to upregulated genes and “-1” assigned to downregulated genes. A Pearson correlation coefficient was calculated for this standard vector versus the vector of median-centered gene expression for each patient. Samples were associated with the Active subtype if the Pearson correlation coefficient was greater than zero; and with the Inactive subtype if the coefficient was less than zero.

### Extraction and metabolomics analysis

Tissues were prepared for extractions, metabolomics data collection and preprocessing by the NIH Eastern Regional Comprehensive Metabolomics Resource Core (ERCMRC). The details regarding sample extraction, data collection, and quality control by the ERCMRC have been described previously [7]. Briefly, samples were thawed on ice in a cold room (4°C) and extracted using 50% acetonitrile in water and homogenized on a MagNA Lyser to generate 0.1 mg/μL supernatants. Quality control (QC) samples were created by pooling a small aliquot from all samples (Total Pool 1). A second QC pool sample was created using unequal volumes of samples (Total Pool 2). Samples were chemically derivatized after a D_2_O exchange and analyzed by GC-TOF-MS (Agilent 7890 gas chromatogram and Leco Pegasus 4D time of flight mass spectrometer). The run order was randomized and samples were analyzed in four batches over 4 separate days. The Total Pool 1 QC sample was analyzed between every 6 sample injections in each batch. The Total Pool 2 QC sample was injected multiple times at the beginning of each batch to condition the column and the system. The GC-MS data from each sample was normalized to its total signal intensity. Analyte peak variations of the pooled samples were determined, and those peaks with a coefficient of variation (CV) > 0.3 were removed from subsequent analysis. As the data was collected over a four-day period, and preprocessing took account of the day-to-day variability from the GC-TOF-MS by adjusting the peaks from each sample by the median of the value from that of the same peak in the Total Pool 1 samples which were run each day. All raw metabolomics data is publicly available at the NIH Common Fund Metabolomics Data Repository and Coordinating Center website, Metabolomics Workbench: http://www.metabolomicsworkbench.org/data/DRCCMetadata.php?Mode=Study&StudyID=ST000054&StudyType=MS&ResultType=1.

Unsupervised hierarchical clustering analysis (average-linkage) by Cluster 3.0 was used to determine subgroups based on metabolomics profiles. The results were visualized using Java Treeview. The metabolites significantly different across clusters were identified by supervised multivariate analysis using orthogonal partial least squares discriminant analysis (OPLS-DA) in SIMCA 13.0 (Umetrics, Umeå, Sweden). Metabolites were library-matched to the Fiehn library [8] and rank-ordered based on the value of the Variable Influence on Projection (VIP ≥ 1). Functional pathway mapping of the known (library-matched) important metabolites was performed using GeneGo (MetaCore-Clarivate Analytics, Philadelphia, PA).

### Statistical and pathway analyses

Associations of metabolomics subgroup with age (<40 years, 40-<60 years, or ≥ 60 years), race (white, African American, or others), body mass index (BMI, <30 kg/m^2^ or ≥30 kg/m^2^), tissue type (cancer-adjacent normal tissue or normal tissue), histological adipose composition (<51% or ≥51%, 51% = median), and Active/Inactive genomic subtype were evaluated qualitatively by Chi-square test or Fisher’s exact test, and quantitatively by odds ratio and corresponding 95% confidence interval (CI) estimated using logistic regression. For normal samples from breast cancer patients, the association of metabolomics subgroup with tumor ER status (negative or positive) and the distance to tumor (near, <1cm; or far, >4cm) were also assessed. Probability values of less than 0.05 were considered statistically significant. The analyses were performed using SAS version 9.4 (SAS Institute).

## Results

Table 1 describes the characteristics of the NBS participants. Normal breast biospecimens (n = 83) from breast cancer patients equaled 84% and 16% (n = 16) were from non-breast cancer patients (reduction mammoplasty). The majority of the participants were white (72%, n = 68), parous (84%, n = 80), and younger than 60 years (72%, n = 71). The other 41% (n = 40) were premenopausal and 47% (n = 47) were obese.

**Table 1:** Associations with sample characteristics, the Active/Inactive subtype, and histological composition.

	Total n = 99	Cluster 1 n = 57	Cluster 2 n = 42	OR (95% CI)	P-value
**Age (year)**					
Age ≥ 60	28 (28)	13 (23)	15 (36)	4.33 (1.14, 16.36)	0.08
40 ≤ Age < 60	52 (53)	29 (51)	23 (55)	2.97 (0.87, 10.19)	
Age < 40	19 (19)	15 (26)	4 (10)	1	
**Menopausal status**					
Premenopausal	40 (41)	25 (44)	15 (37)	0.74 (0.32, 1.68)	0.47
Postmenopausal	58 (59)	32 (56)	26 (63)	1	
**Race**					
African-American	27 (28)	16 (30)	11 (27)	0.87 (0.35, 2.15)	0.76
White	68 (72)	38 (70)	30 (73)	1	
**BMI (kg/m**^**2**^**)**					
Obese (BMI ≥ 30)	47 (47)	22 (39)	25 (60)	2.34 (1.40, 5.28)	0.04
Nonobese (BMI < 30)	52 (53)	35 (61)	17 (40)	1	
**Parity**					
Nulliparous	14 (15)	10 (19)	4 (10)	0.49 (0.14, 1.69)	0.38
Parous	80 (85)	44 (81)	36 (90)	1	
**Tissue type**					
Cancer Adjacent Normal	83 (84)	48 (84)	35 (83)	0.94 (0.32, 2.76)	0.91
Reduction/Prophylactic	16 (16)	9 (16)	7 (17)	1	
Composition epithelium*					
≥median	49 (49)	28 (49)	21 (50)	1.04 (0.47, 2.30)	1.0
< median	50 (51)	29 (51)	21 (50)	1	
Composition stroma*					
≥median	49 (49)	41 (72)	8 (19)	0.09 (0.04, 0.24)	<0.01
< median	50 (51)	16 (28)	34 (81)	1	
Composition adipose*					
≥median	50 (51)	14 (25)	36 (86)	18.43 (6.42, 52.86)	<0.01
< median	49 (49)	43 (75)	6 (14)	1	
**Active/Inactive subtype**					
Active	50 (51)	22 (39)	28 (67)	3.18 (1.38, 7.33)	<0.01
Inactive	49 (49)	35 (61)	14 (33)	1	
**ER status**^**†**^					
Positive	27 (51)	14 (44)	13 (62)	2.09 (0.68, 6.43)	0.20
Negative	26 (49)	18 (56)	8 (38)	1	
**Distance from tumor**^†^**(cm)**					
<3	34 (59)	17 (61)	17 (57)	0.85 (0.30, 2.41)	0.75
<1	24 (41)	11 (39)	13 (43)	1	

*Epithelium Median = 7.2% Stroma Median = 36.2% Adipose Median = 50.9%.

^†^ Among samples from breast cancer patients. 46 patients without ER information and 41 patients with the distance of normal biospecimen to tumor between 1 and 4 cm were not included the ER and distance analysis respectively.

### Identification and interpretation metabolomics-derived subgroups

**Fig 1:**
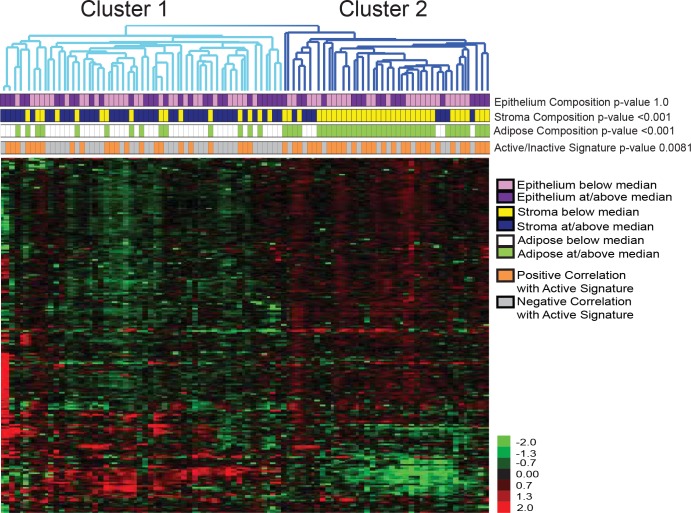
Unsupervised cluster analysis of 220 metabolites across the normal and cancer-adjacent normal breast tissue samples. Pink rectangles represent the samples that have epithelium percent area below the median (7%) for the data set and purple rectangles represent samples that have percent area at or above the median. Yellow rectangles represent the samples that have stroma percent area below the median (36%) for the data set and blue rectangles represent samples that have percent area at or above the median. White rectangles represent the samples that have adipose percent area below the median (51%) for the data set and green rectangles represent samples that have adipose percent area at or above the median for the dataset. Grey rectangles represent the samples that have negative correlation with the active genomic signature and orange rectangles represent samples that have a positive correlation with the active signature.

**Fig 2:**
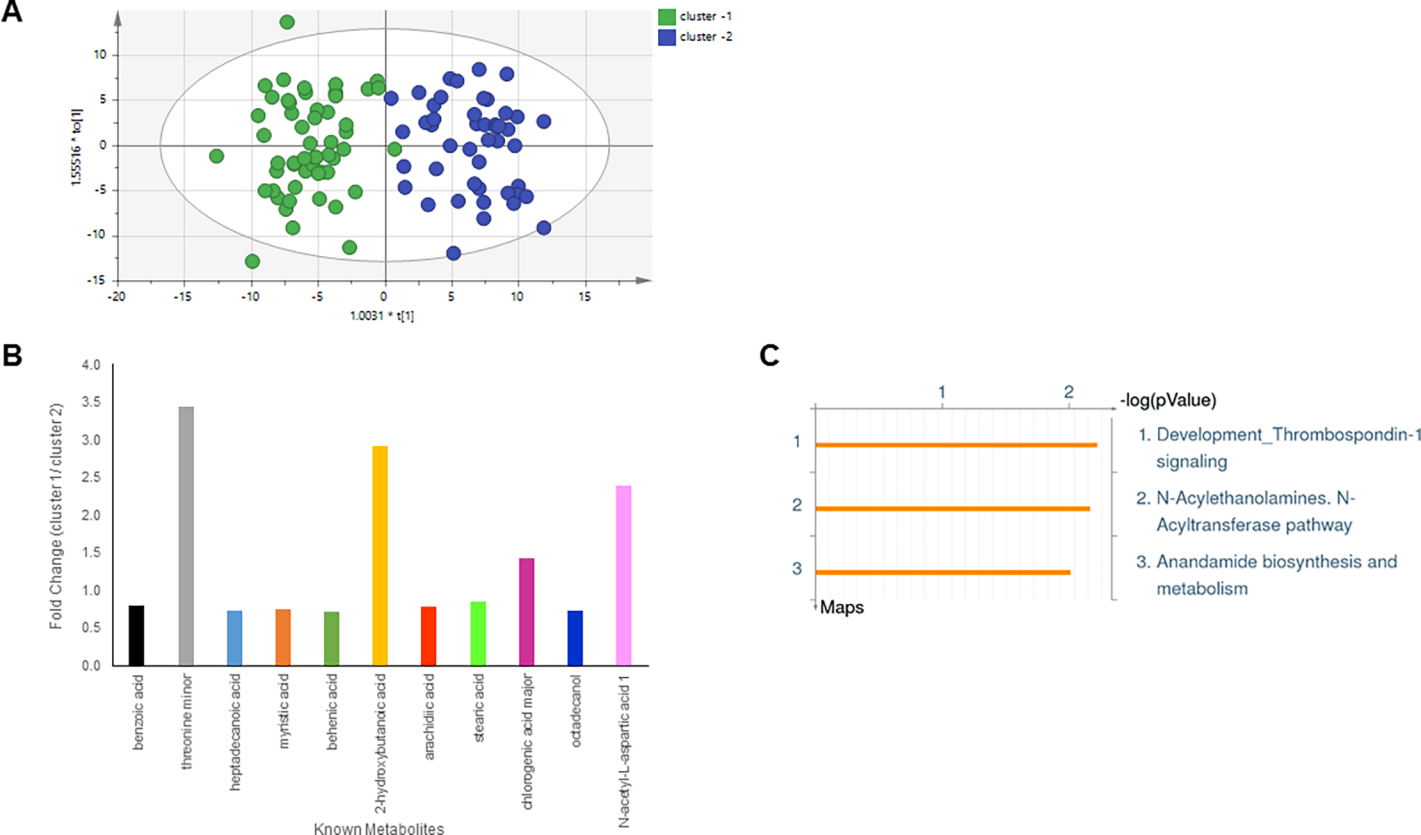
Metabolic Cluster 1 versus Cluster 2. (A) Supervised multivariate analysis (OPLS-DA) of GC-MS generated broad spectrum metabolomics data from non-malignant/cancer-adjacent normal breast tissues. (B) Fold change differences of 11 library-matched metabolites that significantly distinguish metabolic Cluster 1 from Cluster 2. (C) Pathway analysis was done in GeneGo, using the known Cluster 1 –Cluster 2 differentiating metabolites to identify important perturbed biological pathways related to Cluster 1 and Cluster 2 differences.

Unsupervised hierarchical clustering of 220 differential metabolites grouped the tissue samples into two metabolomic clusters (Cluster 1, n = 57; Cluster 2, n = 42) (Fig 1). No differences were detected in biospecimen type (breast cancer-adjacent normal tissue samples: 84% in Cluster 1, 83% in Cluster 2). Supervised multivariate analysis using SIMCA (OPLS-DA) showed a clear separation of the two clusters based on metabolic profile (Fig 2A), and identified 102 metabolites based on VIP ≥ 1, that differentiated Cluster 1 and Cluster 2 (S1 Table) with 11 known metabolites identified after library-matching to the Fiehn library. The fold-difference of the known metabolites was calculated and three (threonine minor, 2-hydroxybutanoic acid, N-acetyl-L-aspartic acid 1) had a fold change greater than 2, when comparing Cluster 1 to Cluster 2 (Fig 2B). Enrichment of endogenous pathways were mapped using all 11 library-matched metabolites in GeneGo (MetaCore). Fig 2C pictorially represents the most significantly enriched pathways, based on the Cluster 1/Cluster 2 fold-change differences and *p*-value thresholds listed in S1 Table.

### Associations with participant characteristics, the Active/Inactive genomic signatures and histological adipose composition

Compared with women in Cluster 1, women in Cluster 2 were older (≥60 years, OR = 4.33, 95% CI = 1.14–16.36) and more obese (OR = 2.34, 95% CI = 1.40–5.28) (Table 1). Tumors of breast cancer patients in Cluster 2 tended to be estrogen receptor (ER) positive (OR = 2.09, 95% CI = 0.68–6.43). No associations were found between metabolomics subtype and race, menopausal status, parity, or distance to tumor.

Previous research with mRNA and microRNA profiles of these samples demonstrated that there are two main phenotypes of breast tissue, termed Active and Inactive [4]. We evaluated the correlation of these two genomic phenotypes and the metabolomics-derived subtypes, and found a strong correlation between Cluster 2 and Active subtype (OR = 3.18, 95% CI = 1.38–7.33, p<0.01) (Fig 1 and Table 1).

Obesity and Active/Inactive gene expression subtype are both associated with tissue composition [5], so we hypothesized that adipose content would also vary by metabolomics-derived phenotype. Here, we observed that 86% of the samples in Cluster 2 had high percent adipose content (defined as ≥ the median of 51%), compared to 25% of the samples in Cluster 1 (OR = 18.43, 95% CI = 6.42–52.86, p<0.01) (Fig 1 and Table 1). Significant reverse association was observed with stromal contents (OR = 0.09, 95%CI = 0.04–0.24, p<0.01), but not with epithelial content (OR = 1.04, 95%CI = 0.47–2.30, p = 1.00).

## Discussion

Using unsupervised clustering analysis, we identified two main metabolic clusters in normal breast. Cluster 2, in which the identified metabolites are suggested to play a role in cell death and molecular transport (Fig 2B), was significantly associated with the Active genomic subtype, as well as obesity and high adipose content in breast tissue.

By using integrated data we demonstrated that multi-layer biological changes are contributing to the breast microenvironment. Brauer et al., previously reported metabolic changes in cancer adjacent normal microenvironment [1]; while in this study, we characterized these changes by defining two distinct metabolic subtypes, and further linked these subtypes to non-malignant genomic breast signatures (Active/Inactive) and tissue adipose content. The Troester lab has previously evaluated the Active/Inactive genomic subtypes for association with mammographic density (MD) and breast tissue composition, and found strong correlations among the Active subtype, high tissue adipose content, and low MD [4, 5]. The current study confirms these previous findings, and extends this correlation circle by adding metabolic Cluster 2 subtype. These consistent findings through integrative data approaches strongly suggest the existence of two broad microenvironmental types of breast tissue. A further question that remains to be addressed is how these microenvironment-defined subtypes mediate an association between risk/prognostic factors, breast cancer occurrence/recurrence and survival outcome, which is very important to understand their role in breast cancer etiology and to identify novel biomarkers to optimize risk prediction and treatment.

Obesity has a dichotomous association with breast cancer, where it has been shown protective in premenopausal women [9], but is an established risk/prognostic factor of breast cancer in postmenopausal women, with various mechanisms proposed to explain its effect on tumor initiation and progression [10–12]. Our data show that obesity affects the breast histological composition, and is associated with genomic and metabolic changes in histologically normal breast [5, 13], suggesting that obesity profoundly alters the breast microenvironment. In terms of metabolomics profiling, we found that the metabolites higher in Cluster 2 compared to Cluster 1 may be involved in pathways related to lipid metabolism through endocannabinoid-related systems that can modulate cancer cell growth and proliferation (N-Acylethanolamines and Anandamide biosynthesis) [14, 15], and more significantly, creating an environment for increased migratory potential (Thrombospondin 1) [16] (Fig 2C). These findings are in line with previous data, where broad changes in lipid metabolism during breast carcinogenesis have been associated with maintaining cell growth, playing a role in cell survival and contributing to metastasis [17–21]. Our analyses describe the overall correlations of obesity with metabolic and genomic profiles in the normal and cancer-adjacent normal breast microenvironment. However, how these changes at each level interact with one another (e.g. network linkage of specific metabolite(s) to certain gene(s)) needs further study.

Age is the strongest risk factor for breast cancer, and is associated with genomic and histological alternations [6, 22–24]. In this study we observed a significant association between metabolic subtype, when comparing young women (<40 years) and old women (≥60 years) (OR = 4.33, 95% CI = 1.14–16.36). This finding is consistent with the data that age is positively associated with the Active genomic signature and high adipose content. However, given this correlation between age and obesity and the limited sample size of our study, these data will not allow us to test whether their effect on microenvironment is independent, and if not, how age and obesity interact across microenvironmental changes.

Several studies have evaluated the association between tumor characteristics (such as molecular subtypes and ER status) and metabolomics profiles of breast tissues. Some investigators observed a separation between ER positive and ER negative tumors based on metabolomics profiles [7, 17, 25], whereas others showed overlapping patterns between ER positive and ER negative samples [1, 26]. In the current study, we observed that metabolic Cluster 2 included more women with ER positive tumors (OR = 2.09). However, our findings should be interpreted with caution in that it was based on univariate analysis (not adjusting for potential confounders, e.g. obesity), and not statistically significant (p = 0.20).

Our study should be considered in light of limitations and strengths. First, the sample size of our study may lead to imprecise effect estimates (e.g. OR of adipose composition) and lack of statistical power for multivariate analyses or stratified analyses. Future studies with larger sample size are needed to overcome these caveats and validate our findings. Second, although we detected over a hundred significant compound ions, only 11 could be library-matched to known metabolites for use in pathway analysis. Therefore, the results of pathway analysis do not characterize the two metabolic Clusters comprehensively. One important strength of our study is the use of normal and cancer-adjacent normal breast tissue. Most metabolic studies are done in the diseased tissue/tumor where the characteristics of the pathologic tissue are compared to normal tissue in order to understand the variations from normalcy that accounts for the abnormality manifested as disease/pathology [26, 27]. However, the corresponding changes in normal versus cancer-adjacent normal tissue are not well understood. In our study, we used both normal and cancer-adjacent normal breast tissue samples from the Normal Breast Study (NBS), and provided significant associations between genomic, histologic, and metabolic profiles within a population of women with histologically normal-appearing breast tissue. Our results help to increase understanding on how normal tissue responds to breast cancer risk factor exposures, the adjacent tumor microenvironment, and how these dynamics may mediate breast cancer risk and outcome.

To identify targetable pathways to disrupt the process of tumor growth and progression, future research with sufficient sample size of normal breast tissue is needed to better characterize genomic, metabolomic and histological microenvironments, and to understand how these associated risk factors (e.g. obesity) drive breast tissue microenvironment alterations and ultimately promote disease. In addition, the identification and analysis of additional metabolites that may have a role in modulating the effect of various risk factors will help to further our understanding of normal and cancer-adjacent normal breast phenotypes.

## Supporting information

S1 Table Significant Cluster 1 vs. Cluster 2 metabolites. (DOCX)
