# Supplementary material for: Correlated metabolomic, genomic, and histologic phenotypes in histologically normal breast tissue
Source: PLoS One. 2018 Apr 18;13(4):e0193792. doi: 10.1371/journal.pone.0193792 (PMC5905995; doi:10.1371/journal.pone.0193792)
Supplement: S1 Table — (DOCX) [file pone.0193792.s001.docx]

**Supporting Information**

**S1 Table**. Significant Cluster 1 vs. Cluster 2 metabolites.

| **Metabolite/**  **Unknown Ion ID** | **VIP** | ***p*-value** | **Fold Change**  **(C1/C2)** | | **FDR** | |
| --- | --- | --- | --- | --- | --- | --- |
| 203294 | 1.64 | 0.3091 | 0.7175 | 0.3488 | |  |
| 1644 | 1.61 | 0.0000 | 0.6747 | 0.0000 | |  |
| 203063 | 1.61 | 0.2007 | 2.8199 | 0.2426 | |  |
| 705 | 1.59 | 0.0000 | 0.7428 | 0.0000 | |  |
| 202861 | 1.53 | 0.1169 | 1.7446 | 0.1504 | |  |
| 202771 | 1.53 | 0.1070 | 0.7263 | 0.1402 | |  |
| 203047 | 1.52 | 0.1913 | 1.8343 | 0.2325 | |  |
| 34560 | 1.51 | 0.0000 | 0.7091 | 0.0001 | |  |
| 202650 | 1.51 | 0.0646 | 2.2750 | 0.0893 | |  |
| 203086 | 1.51 | 0.2141 | 0.5904 | 0.2519 | |  |
| **benzoic acid** | 1.50 | 0.4489 | 0.7898 | 0.4841 | |  |
| 148177 | 1.47 | 0.0018 | 0.7559 | 0.0032 | |  |
| 63712 | 1.47 | 0.0002 | 0.6852 | 0.0003 | |  |
| 1447 | 1.46 | 0.0000 | 0.6875 | 0.0000 | |  |
| 63607 | 1.46 | 0.0001 | 0.7523 | 0.0003 | |  |
| 1455 | 1.45 | 0.0000 | 0.6560 | 0.0000 | |  |
| 26 | 1.45 | 0.0000 | 0.7960 | 0.0000 | |  |
| 296 | 1.45 | 0.0000 | 0.6773 | 0.0000 | |  |
| 40961 | 1.44 | 0.0001 | 0.7654 | 0.0002 | |  |
| 203118 | 1.44 | 0.2426 | 0.6903 | 0.2824 | |  |
| 73 | 1.43 | 0.0000 | 1.5262 | 0.0000 | |  |
| 203065 | 1.42 | 0.2050 | 2.5112 | 0.2451 | |  |
| **threonine minor** | 1.41 | 0.9092 | 3.4346 | 0.9134 | |  |
| 1433 | 1.40 | 0.0000 | 2.4215 | 0.0000 | |  |
| 1496 | 1.40 | 0.0000 | 0.7302 | 0.0000 | |  |
| **heptadecanoic acid** | 1.40 | 0.4832 | 0.7247 | 0.5111 | |  |
| 202880 | 1.39 | 0.1204 | 0.6449 | 0.1540 | |  |
| 1468 | 1.39 | 0.0000 | 0.6406 | 0.0000 | |  |
| 34655 | 1.38 | 0.0001 | 0.6633 | 0.0002 | |  |
| 168 | 1.36 | 0.0000 | 0.7369 | 0.0000 | |  |
| 1459 | 1.35 | 0.0000 | 0.6790 | 0.0000 | |  |
| 186 | 1.34 | 0.0000 | 0.7318 | 0.0000 | |  |
| 47 | 1.34 | 0.0000 | 4.6275 | 0.0000 | |  |
| **myristic acid** | 1.34 | 0.5533 | 0.7480 | 0.5824 | |  |
| 1603 | 1.34 | 0.0000 | 0.7620 | 0.0000 | |  |
| 3305 | 1.33 | 0.0000 | 0.7585 | 0.0000 | |  |
| 113589 | 1.33 | 0.0008 | 0.7656 | 0.0015 | |  |
| **behenic acid** | 1.33 | 0.4253 | 0.7117 | 0.4609 | |  |
| 1460 | 1.32 | 0.0000 | 0.7535 | 0.0000 | |  |
| **2-hydroxybutanoic acid** | 1.32 | 0.4204 | 2.9230 | 0.4601 | |  |
| **arachidiic acid** | 1.31 | 0.4229 | 0.7735 | 0.4606 | |  |
| 2779 | 1.31 | 0.0000 | 0.7934 | 0.0000 | |  |
| 36066 | 1.29 | 0.0001 | 0.6793 | 0.0002 | |  |
| 1454 | 1.29 | 0.0000 | 0.6428 | 0.0000 | |  |
| 150 | 1.29 | 0.0000 | 0.7310 | 0.0000 | |  |
| 150498 | 1.28 | 0.0069 | 0.7384 | 0.0111 | |  |
| 202811 | 1.28 | 0.1111 | 0.6812 | 0.1447 | |  |
| 163955 | 1.27 | 0.0181 | 0.7756 | 0.0276 | |  |
| 479 | 1.27 | 0.0000 | 0.7556 | 0.0000 | |  |
| 1491 | 1.26 | 0.0000 | 0.5723 | 0.0000 | |  |
| 1579 | 1.26 | 0.0000 | 0.7588 | 0.0000 | |  |
| 1457 | 1.26 | 0.0000 | 0.7451 | 0.0000 | |  |
| 144 | 1.26 | 0.0000 | 0.7279 | 0.0000 | |  |
| 203466 | 1.25 | 0.3843 | 0.8807 | 0.4227 | |  |
| 202653 | 1.25 | 0.0653 | 0.7335 | 0.0898 | |  |
| 1617 | 1.25 | 0.0000 | 0.6543 | 0.0000 | |  |
| 2726 | 1.24 | 0.0000 | 0.7603 | 0.0000 | |  |
| 113585 | 1.23 | 0.0005 | 0.7608 | 0.0010 | |  |
| 114272 | 1.23 | 0.0011 | 0.8088 | 0.0020 | |  |
| 202924 | 1.23 | 0.1484 | 0.7451 | 0.1865 | |  |
| 1513 | 1.22 | 0.0000 | 0.7465 | 0.0000 | |  |
| 202673 | 1.22 | 0.0787 | 0.7390 | 0.1075 | |  |
| 161368 | 1.22 | 0.0113 | 0.7630 | 0.0176 | |  |
| 1506 | 1.21 | 0.0000 | 0.7571 | 0.0000 | |  |
| 232 | 1.21 | 0.0000 | 0.8050 | 0.0000 | |  |
| 1430 | 1.20 | 0.0000 | 0.7943 | 0.0000 | |  |
| 202704 | 1.20 | 0.0972 | 0.7354 | 0.1297 | |  |
| 202885 | 1.19 | 0.1302 | 0.8245 | 0.1656 | |  |
| 113577 | 1.16 | 0.0004 | 0.7633 | 0.0008 | |  |
| 202915 | 1.16 | 0.1363 | 0.7609 | 0.1723 | |  |
| 174 | 1.16 | 0.0000 | 0.8436 | 0.0000 | |  |
| 203081 | 1.15 | 0.2119 | 2.9486 | 0.2506 | |  |
| 203361 | 1.15 | 0.3653 | 0.7210 | 0.4059 | |  |
| 202971 | 1.14 | 0.1653 | 0.8000 | 0.2054 | |  |
| 51 | 1.14 | 0.0000 | 0.7126 | 0.0000 | |  |
| 2202 | 1.14 | 0.0000 | 2.4546 | 0.0000 | |  |
| 203247 | 1.13 | 0.2792 | 0.8200 | 0.3199 | |  |
| 3359 | 1.13 | 0.0000 | 0.7301 | 0.0000 | |  |
| 1481 | 1.12 | 0.0000 | 0.8139 | 0.0000 | |  |
| 58 | 1.11 | 0.0000 | 0.7840 | 0.0000 | |  |
| 49 | 1.11 | 0.0000 | 0.7979 | 0.0000 | |  |
| 202723 | 1.11 | 0.1005 | 2.2270 | 0.1332 | |  |
| 89 | 1.11 | 0.0000 | 1.5792 | 0.0000 | |  |
| 166362 | 1.10 | 0.0364 | 1.6732 | 0.0523 | |  |
| 1557 | 1.10 | 0.0000 | 0.8160 | 0.0000 | |  |
| 203328 | 1.10 | 0.3602 | 0.7420 | 0.4022 | |  |
| 14 | 1.09 | 0.0000 | 2.3202 | 0.0000 | |  |
| 122691 | 1.09 | 0.0014 | 0.8361 | 0.0025 | |  |
| 35455 | 1.07 | 0.0001 | 0.8225 | 0.0002 | |  |
| **stearic acid** | 1.07 | 0.9046 | 0.8517 | 0.9129 | |  |
| 10109 | 1.06 | 0.0000 | 2.2091 | 0.0001 | |  |
| **chlorogenic acid major** | 1.06 | 0.4775 | 1.4197 | 0.5099 | |  |
| 3745 | 1.06 | 0.0000 | 0.8102 | 0.0000 | |  |
| 1621 | 1.06 | 0.0000 | 0.7509 | 0.0000 | |  |
| **octadecanol** | 1.04 | 0.6133 | 0.7254 | 0.6395 | |  |
| 113636 | 1.04 | 0.0008 | 0.7659 | 0.0016 | |  |
| 113887 | 1.03 | 0.0009 | 0.8875 | 0.0016 | |  |
| 63674 | 1.03 | 0.0002 | 0.8603 | 0.0003 | |  |
| 150584 | 1.03 | 0.0081 | 0.8296 | 0.0130 | |  |
| **N-acetyl-L-aspartic acid 1** | 1.01 | 0.6015 | 2.3899 | 0.6301 | |  |
| 170217 | 1.00 | 0.0406 | 1.8532 | 0.0572 | |  |
| 63606 | 1.00 | 0.0001 | 0.7826 | 0.0003 | |  |

This table highlights metabolites that significantly (variable importance in projection (VIP ≥ 1) differ between clusters 1 and 2 in supervised multivariate analysis (OPLS-DA) via GC-MS, and ranked in order using VIP. C1 is Cluster 1 and C2 is Cluster 2. FDR is false discovery rate. Bold-faced library-matched metabolites were used in GeneGo pathway mapping analysis.
